# A Hospital for Lepers in Iceland

**Published:** 1895-12-28

**Authors:** 


### The Institutional Workshop.

### A HOSPITAL FOR LEPERS IN ICELAND.

The Icelandish Government has framed two proposals dealing with the leprosy of that island; the first refers to the building of a separate hospital for lepers, the other deals with the isolation of lepers, &c. It is proposed to erect a hospital at Reykjavik capable of holding sixty leprous patients. The number of lepers in Iceland cannot, it is thought, be estimated at less than two hundred, or three in every 1,000, which is considerably higher than what was the case in Norway some thirty or forty years ago, when the disease was looked upon as comparatively frequent. There are no exact statistics as to the lepers, but the thirty-three State-subsidised doctors in Iceland have received instructions to procure an accurate and complete list of Icelandish lepers by July 1st, 1898. The hospital is calculated to cost 62,000kr., and is in the first instance intended to receive gratis such patients as the authorities desire should be treated there. Other lepers, who might be wishful to be admitted into the hospital, can be received subject to an annual payment. The other proposal, dealing with isolation of lepers, &c., is to a great extent framed in accordance with the Norwegian law. Lepers receiving money from the guardians of the poor can for the future be transferred to the hospital should the municipal authorities desire this to be done. Should the latter not wish this, they can be placed in separate premises, or be taken care of in a manner which the district doctor may deem satisfactory. Husband and wife must not be separated unless circumstances make this absolutely necessary. Patients who do not receive poor aid can under certain circumstances be made to enter a hospital—a rule which does not exist in Norway. This can be done when the patient does not comply with the regulations for isolation, cleanliness, &c., or when the district doctor considers that the nature of the disease or the danger of infection may demand such a step to be taken. The removal of the patient to the hospital is done after a consultation amongst the various authorities. In such cases, too, husband and wife must not be separated unless a stringent necessity for doing so exists. Premises that have been occupied, and bedding, clothing, &c., that have been used by lepers, must be efficiently disinfected—a point which in a country like Iceland, where the distances are great and the means of communication inadequate, may cause a good deal of trouble.

